# A Man with Sudden Onset Leg Pain and Weakness

**DOI:** 10.5811/cpcem.20330

**Published:** 2024-08-26

**Authors:** James DeChiara, Lisa Skinner

**Affiliations:** * Madigan Army Medical Center, Department of Emergency Medicine, Joint Base Lewis-McChord, Washington; † Providence St. Peter Hospital, Olympia, Washington

**Keywords:** embolism, aortic occlusion, embolectomy, thrombosis

## Abstract

**Case Presentation:**

An 89-year-old male who had been holding dabigatran in the setting of transcarotid artery revascularization presented to the emergency department with sudden onset leg pain and weakness. Computed tomography angiography revealed acute aortic occlusion and thrombosis of the bilateral common iliac arteries. He underwent aortoiliac and femoral embolectomies and stenting of the bilateral common iliac arteries and returned to his baseline functional status.

**Discussion:**

Acute aortic occlusion is a rare but often devastating vascular emergency characterized by obstruction of the aorta by an embolus or thrombosis. Diagnosis can be challenging as it may be mistaken for spinal pathology, which can lead to delays in diagnosis. Despite advances in diagnostic modalities and interventions, acute aortic occlusion often results in high rates of major morbidity and mortality.

## CASE PRESENTATION

An 89-year-old male with a history of atrial fibrillation and left transcarotid artery revascularization (TCAR) four days prior presented to the emergency department with sudden onset pain and weakness in the bilateral lower extremities. His pain progressed to involve the lower back, and he began having extremity numbness and tingling. He was taking aspirin and clopidogrel but was holding his previously prescribed dabigatran in the setting of recent TCAR. His physical exam revealed abdominal tenderness, absent dorsalis pedis pulses bilaterally, and profound weakness and loss of sensation of bilateral lower extremities. Due to the rapidity of onset and lack of dorsalis pedis pulses, a vascular etiology was suspected.

**Image 1:**
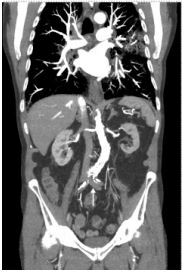
Coronal computed tomography angiography abdomen-aorta demonstrating thrombosis of the aorta extending into the iliac arteries (arrow).

**Image 2:**
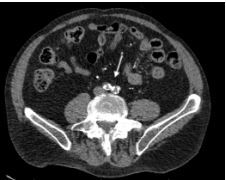
Axial computed tomography angiography abdomen-aorta showing bilateral occlusion of the iliac arteries (arrow).

Computed tomography angiography (CTA) chest abdomen and pelvis, and CTA abdomen-aorta with bilateral femoral runoff were ordered, which revealed acute aortic occlusion and thrombosis of the bilateral common iliac arteries (Images 1 and 2). The patient subsequently underwent aortoiliac and femoral embolectomies and stenting of the bilateral common iliac arteries. He was transferred to the intensive care unit postoperatively, where he recovered and was extubated on day two. He was transferred to the acute care service on day four and discharged after achieving independent ambulation on day nine.

## DISCUSSION

Acute aortic occlusion occurs due to thrombosis, embolus, or occluded grafts or stent, with in situ thrombosis accounting for approximately 64–72% of cases.^1^,^2^ Mortality rates are reported as 21–52%.^1^,^3^ Factors that increase the risk of aortic occlusion include peripheral arterial disease, smoking, and hypercoagulable states, among others. Physical exam findings can include lower extremity tenderness and neurologic deficits such as paralysis, weakness, and sensory deficits, which more frequently occur unilaterally.^2^ Abdominal pain and tenderness may suggest clot burden and occlusion above the level of the iliac bifurcation. Diminished or absent distal pulses, and the presence of cool or mottled skin can help differentiate aortic occlusion from neurologic pathology.

While ultrasonography may show an echogenicity in the vessel lumen and can be performed rapidly at the bedside,^4^ CTA is the test of choice for definitive diagnosis. Management of acute aortic occlusion includes anticoagulation and emergent revascularization.^3^ Most commonly, the surgery of choice is a thromboembolectomy, followed by thrombolysis and axillobifemoral or aortobifemoral bypass; however, this is dependent on the etiology of occlusion. While embolectomy is more common in patients with an underlying embolic occlusion, bypass surgery has been the procedure of choice for patients with in situ thrombosis.^1^,^2^

**CPC-EM Capsule**

What do we already know about this clinical entity?

*Acute aortic occlusion typically presents with lower extremity motor and sensory deficits requiring emergent revascularization, yet still has high rates of mortality.*

What is the major impact of the image(s)?

*CT angiography abdomen-aorta with bilateral femoral runoff should be considered in patients who present with acute onset, lower extremity neurovascular deficits.*

How might this improve emergency medicine practice?

*Clinicians should consider vascular etiologies when developing a differential diagnosis for patients presenting with symptoms that raise concern for spinal pathology.*
